# Late Maternal Deaths and Deaths from Sequelae of Obstetric Causes in the Americas from 1999 to 2013: A Trend Analysis

**DOI:** 10.1371/journal.pone.0160642

**Published:** 2016-09-14

**Authors:** Federico G. de Cosio, Safia S. Jiwani, Antonio Sanhueza, Patricia N. Soliz, Francisco Becerra-Posada, Marcos A. Espinal

**Affiliations:** Pan American Health Organization, Washington D. C., United States of America; Stellenbosch University, SOUTH AFRICA

## Abstract

**Background:**

Data on maternal deaths occurring after the 42 days postpartum reference time is scarce; the objective of this analysis is to explore the trend and magnitude of late maternal deaths and deaths from sequelae of obstetric causes in the Americas between 1999 and 2013, and to recommend including these deaths in the monitoring of the Sustainable Development Goals (SDGs).

**Methods:**

Exploratory data analysis enabled analyzing the magnitude and trend of late maternal deaths and deaths from sequelae of obstetric causes for seven countries of the Americas: Argentina, Brazil, Canada, Colombia, Cuba, Mexico and the United States. A Poisson regression model was developed to compare trends of late maternal deaths and deaths from sequelae of obstetric causes between two periods of time: 1999 to 2005 and 2006 to 2013; and to estimate the relative increase of these deaths in the two periods of time.

**Findings:**

The proportion of late maternal deaths and deaths from sequelae of obstetric causes ranged between 2.40% (CI 0.85% – 5.48%) and 18.68% (CI 17.06% – 20.47%) in the seven countries. The ratio of late maternal deaths and deaths from sequelae of obstetric causes per 100,000 live births has increased by two times in the region of the Americas in the period 2006-2013 compared to the period 1999-2005. The regional relative increase of late maternal death was 2.46 (p<0.0001) times higher in the second period compared to the first.

**Interpretation:**

Ascertainment of late maternal deaths and deaths from sequelae of obstetric causes has improved in the Americas since the early 2000’s due to improvements in the quality of information and the obstetric transition. Late and obstetric sequelae maternal deaths should be included in the monitoring of the SDGs as well as in the revision of the International Classification of Diseases’ 11^th^ version (ICD-11).

## Introduction

Reducing maternal mortality (MM) was a key objective in international development [1], a fundamental component of the Millennium Development Goals (MDGs), and remains a pressing unfinished agenda for the post-2015 Sustainable Development Goals (SDGs) [2]. For the SDGs era, it is proposed that the maternal mortality ratio (MMR) be reduced globally to less than 70 per 100,000 live births by 2030 [3, 4]. However, it is important to note that in the past 20 years, the MMR dropped by 45% globally, 43% in Latin America and 30% in the Caribbean; in fact, none of the countries in the Americas met the fifth MDG of a 75% reduction [2]. The nature of a maternal death, as a relatively rare outcome [5], makes it prone to under-reporting and misclassification [6]. Hence, classifying, capturing and measuring maternal deaths has been a complex issue, even in countries with advanced statistical systems [6, 7]. Nevertheless, MM remains a crucial indicator for measuring human and social development globally [8–10], and the elements that characterize its definition are debated. With the exception of the 2015 estimates developed by the Maternal Mortality Estimation Inter-Agency Group (MMIEG), composed of WHO, UNFPA, UNICEF, the World Bank, and the United Nations Population Division, previous estimates included late maternal deaths and deaths due to obstetric sequelae [3, 11]. This change arose from the fact that Member States of the Americas expressed their concern regarding the inclusion of late and sequelae maternal deaths in MMR estimations through the Pan American Health Organization’s 54^th^ Directing Council held in Washington D.C. in October 2015, documented in resolution CD54.R18. [12]; this has pushed the need for analyses on the trend and magnitude of late maternal deaths in the Americas.

## Background and Significance

The World Health Organization (WHO) defines a maternal death as “*the death of a woman while pregnant or within 42 days of termination of pregnancy*, *irrespective of the duration and the site of the pregnancy*, *from any cause related to or aggravated by the pregnancy or its management but not from accidental of incidental causes*” [13] This definition corresponds to the codes O00-O95 and O98-O99 of the International Classification of Diseases’ 10th revision (ICD-10 volume 2), and therefore excludes deaths from any obstetric causes occurring after 42 days but less than one year after delivery (coded O96), deaths from sequelae of obstetric causes occurring one year or more after delivery (coded O97), as well as deaths related to obstetrical tetanus (coded A34).

Despite advancements in coding and classification of maternal deaths, weak routine reporting systems at national levels [6] coupled with incomplete recording as well as poor data quality and monitoring [14] has pushed the need for more accurate estimates of MM. The MMEIG developed estimation methods addressing the constraints related to the use of reported data, and analyzing MM trends from 1990 onwards [14, 15]; the estimates for the 1990–2013 period included all deaths coded to chapter XV of the ICD-10, including late maternal deaths and deaths from sequelae of obstetric causes (coded O96-O97), in order to maintain comparison with previous data sets [11]. This criterion diverges from the definition of a maternal death as per the ICD-10 volume 2. In light of the constraints around the definition, classification and estimation of MM, adequate assessment of MMR estimates will be a critical issue in evaluating the baseline and monitoring progress of the SDGs.

Although the mortality database of PAHO includes 18 countries that produce data on this topic, the analysis presented focuses on the magnitude of the issue in Argentina, Brazil, Canada, Colombia, Cuba, Mexico and the USA as these are the countries with high quality mortality data on late maternal deaths and deaths due to obstetric sequelae that met the inclusion criteria. It has been believed that these deaths account for 1 to 2% of all maternal deaths globally [11], though this is not the case for the region of the Americas. Our objective is to examine the trend and magnitude of late maternal deaths and deaths from sequelae of obstetric causes between 1999 and 2013.

## Methods

An ecological study was conducted to investigate the trend of late maternal deaths and deaths from sequelae of obstetric causes (ICD-10 codes O96-O97) in the Americas, using the regional mortality database of PAHO between 1999 and 2013. Annual data by country revealed the number of maternal deaths classified as per the ICD-10 codes O96-O97. All women aged 10–54 years old were included in order to capture all deaths related to pregnancy.

For this study, we chose several criteria for inclusion of the countries of the Americas by order of highest weight: (a) reporting of maternal deaths coded O96-O97 during the period 1999–2013; (b) data quality assessed by a mortality under-registration data index no greater than 25 percent, as well as a proportion of ill-defined causes of death no greater than 10%, as assessed in 2014 by the PAHO Basic Health Indicators for the Americas; (c) countries had data for at least 8 years of the 15-year study period. Eighteen countries were initially considered for the analysis, and seven countries met the inclusion criteria: Argentina, Brazil, Canada, Colombia, Cuba, Mexico and the United States of America.

Exploratory data analysis consisted of computing, by country, the proportion of maternal deaths coded as O96-O97 for the periods 1999–2005, 2006–2013 and for the entire 1999–2013 period. Separate analyses were conducted dividing the total period of time into period 1 (1999–2005) and period 2 (2006–2013): the rationale being the evidence on the recent surge in 096–097 coded deaths. Finally, the ratio of O96-O97 coded deaths was computed by country and period of time, in addition to the percent variation in this ratio between the two periods.

A Poisson regression model was used for fitting the number of late maternal deaths and deaths from sequelae of obstetric causes as response and year as the covariate (or independent variable). This model allowed estimating the average annual percentage rate variation (AAPV), as well as confidence intervals and p-values.

In order to compare the periods 2006–2013 and 1999–2005, the Poisson regression model was also used to fit the cases of late maternal deaths and deaths from sequelae of obstetric causes as the response variable and period of time (2006–2013 /1999–2005) as the categorical covariate. The estimated relative increase (equivalent to the well-known relative risk) of late maternal deaths and deaths from obstetric sequelae by period of time, including confidence intervals and p-values were calculated using the 1999–2005 period as a reference. Besides, this model allowed computing the estimated percent of variation of late maternal deaths and deaths from sequelae of obstetric causes between the two periods, with associated confidence intervals and p-values.

All analyses were carried out using Stata version 12.0 (StataCorp. 2011. Release 12. College Station, TX).

## Results

A total of 85,654 maternal deaths (coded O00-O99) were identified among women aged 10–54 years old for the eighteen countries that reported any 096–097 deaths between 1999 and 2013. Among these, 4,417 deaths or 5.10% were reported as late maternal deaths or deaths from sequelae of obstetric causes (O96-O97).

Not all countries of the Americas report O96-O97 deaths for MDG monitoring; among the seven countries that complied with our inclusion criteria, 4,332 deaths or 6.47%, out of 66,975 total maternal deaths (O00-O99), were identified as O96-O97 (Table 1): the highest proportion was reported in the United States (18.68%) followed by Cuba (11.53%). When comparing period of time 1(1999–2005) and period of time 2(2006–2013), all countries reported a higher proportion of O96-O97 deaths in period 2, except Canada (3.87% compared to 6.40%) and Cuba (11.32% compared to 11.82%).

**Table 1:** Proportion of late maternal deaths and deaths from sequelae of obstetric causes (O96-O97) by country, among women aged 10–54 years old.

Country	Period 1: 1999–2005		Period 2: 2006–2013		Total Period: 1999–2013	
	% (95%CI)	n	% (95%CI)	n	% (95%CI)	n
Argentina	5.05 (2.01–10.48)	107	6.68 (3.97–11.60)	175	5.95 (3.80–9.44)	282
Brazil	3.39 (1.94–5.56)	394	5.43 (4.04–7.37)	731	4.48 (3.39–5.81)	1,125
Canada	6.40 (2.24–47.09)	8	3.87 (3.01–56.35)	6	5.0 (1.27–31.47)	14
Colombia	1.54 (0.27–8.10)	66	3.51 (1.34–8.46)	117	2.40 (0.85–5.48)	183
Cuba	11.82 (4.41–25.31)	36	11.32 (5.40–22.97)	52	11.53 (6.29–19.67)	88
Mexico	1.88 (0.60–5.06	170	5.34 (3.67–7.82)	465	3.57 (2.43–5.38)	635
USA	11.05 (8.34–14.52)	398	22.53 (20.55–24.63)	1,607	18.68 (17.06–20.47)	2,005
Total Americas	3.79 (2.86–5.07)	1,179	8.79 (7.85–9.82)	3,153	6.47 (5.77–7.23)	4,332

n = number of deaths

**Fig 1:**
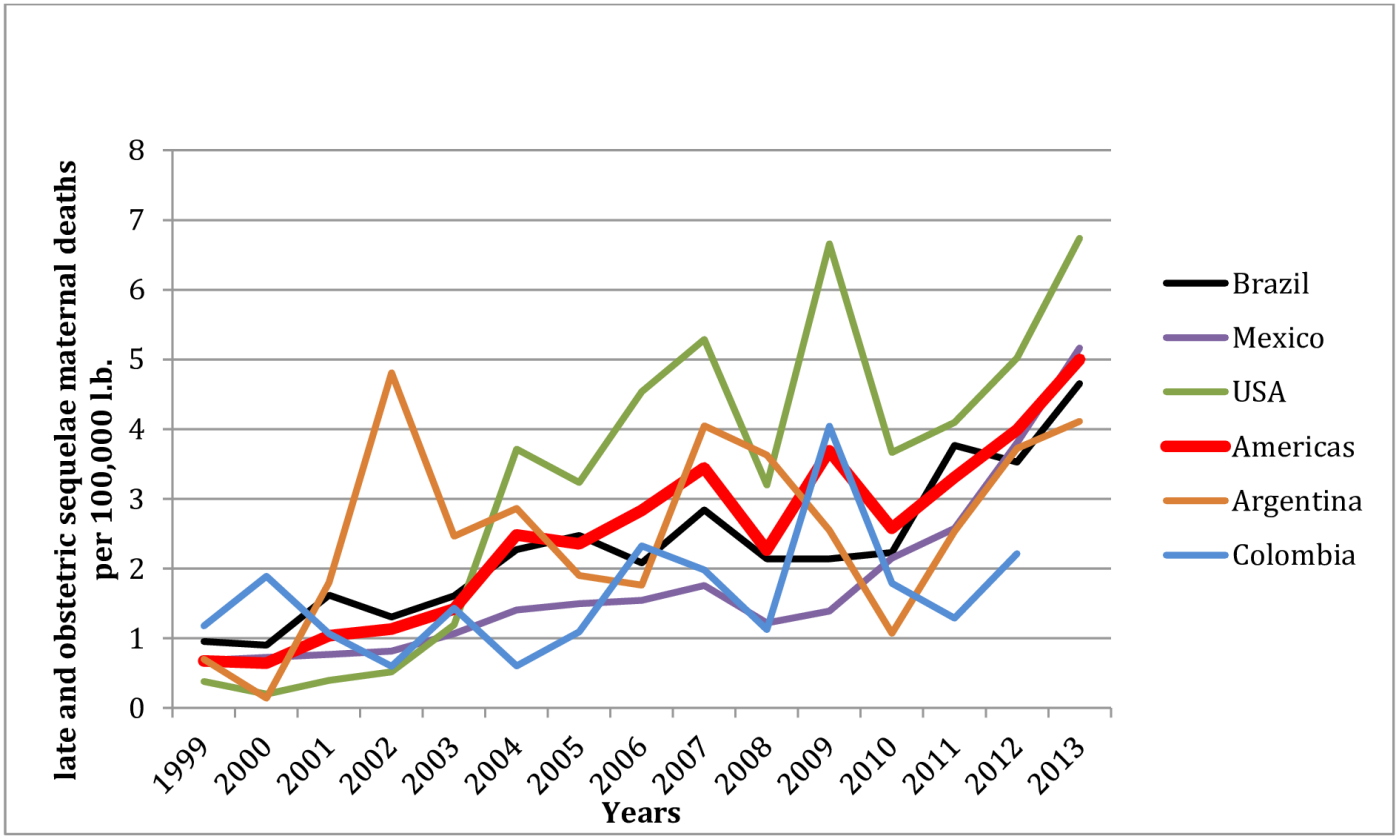
Trend of late and obstetric sequelae maternal mortality ratio: Argentina, Brazil, Colombia, Mexico, USA and the Americas region between 1999 and 2013.

Regionally, the trend in late and obstetric sequelae maternal deaths describes an increasing tendency in the Americas (Fig 1). At the country level, an increased trend of late and sequelae maternal deaths is more noticeable from 2003 onwards in the USA and 2009 and 2010 onwards in Mexico, Brazil and Argentina respectively, with an accentuated rise in the last three years (Fig 1).

The average annual percent variation (AAPV) in late maternal deaths and deaths from sequelae of obstetric causes from 1999 to 2013 indicates a regional increase of 12.4%. The largest difference is observed in the USA (15.4%), followed by Mexico (15.1%) and Brazil (9.7%). (Table 2)

**Table 2:** Average annual percent variation (AAPV) in late maternal deaths and deaths from sequelae of obstetric causes during the years 1999–2013.

		95% Confidence Interval		
Country	Average Annual % Variation	Lower Limit	Upper Limit	p value
Argentina	5.5	2.6	8.5	0.0001
Brazil	9.7	8.2	11.3	<0.0001
Canada	-1.1	-13.8	13.4	0.8711
Colombia	5.9	2.1	9.8	0.0022
Cuba	2.2	-3.2	7.8	0.4332
Mexico	15.1	12.8	17.4	<0.0001
USA	15.4	14.1	16.7	<0.0001
Americas	12.4	11.6	13.2	<0.0001

In the region of the Americas the relative increase (RI) and percent variation of late maternal deaths and deaths from sequelae of obstetric causes is 2.46 times higher in the second period (2006–2013) compared to the first period (1999–2005) (p<0.0001), with an average annual variation of 145.8%. The USA has the highest RI of 3.51, p<0.0001 and an annual variation of 250.7% followed by Mexico (RI = 2.47, p<0.0001) with a percent variation of 147.0% and Brazil (RI = 1.85, p<0.0001) with a percent variation of 84.7% (Table 3). These findings confirm a significant relative increase of late and obstetric sequelae maternal deaths in the Americas between 1999–2005 and 2006–2013, due to increased ascertainment of these deaths resulting from improved quality of maternal mortality information and reduced delays in the delivery of care in the health system; additionally, the obstetric transition state that most of the countries of the Americas are undergoing may further contribute to the relative increase of late and obstetric sequelae maternal deaths, although further research will be needed to confirm this finding [16, 17].

**Table 3:** Relative increase and percent variation of late maternal deaths and deaths from sequelae of obstetric causes between the two periods: 2006–2013 compared to 1999–2005.

		95% Confidence Interval				95% Confidence Interval	
Country	Relative Increase	Lower Limit	Upper Limit	p value	Percent Variation	Lower Limit	Upper Limit
Argentina	1.39	1.10	1.77	0.0069	39.3	9.5	77.2
Brazil	1.85	1.63	2.09	<0.0001	84.7	63.4	108.8
Canada	0.59	0.21	1.71	0.3329	-40.7	-79.4	70.8
Colombia	1.65	1.22	2.23	0.0011	65.3	22.2	123.4
Cuba	1.43	0.94	2.19	0.0969	43.3	-6.3	119.2
Mexico	2.47	2.07	2.94	<0.0001	147.0	107.2	194.5
USA	3.51	3.14	3.91	<0.0001	250.7	214.3	291.4
Americas	2.46	2.30	2.63	<0.0001	145.8	129.9	162.8

## Discussion

The proportion of late maternal deaths and deaths from sequelae of obstetric causes, although believed to be 1–2% globally [11, 15] has risen in recent years in the Americas as shown in our study. Torres and colleagues in Mexico found that an active search for cause of death led to reclassifying 5.7% of maternal deaths as late or from sequelae of obstetric causes [18]. Similarly, in Jamaica, 22 out of 81 pregnancy-related deaths in 2008 captured through vital registration were classified as late maternal deaths [7]; Data from the Brazilian Ministry of Health Information Systems for the period 2008–2011 suggested that 11.29% of deaths were reclassified as late maternal deaths [5]. In Argentina, in a paired case-control study, 22% of deaths in 2001–2002 were classified as late maternal deaths [19]. These data not only revealed the improved capture of maternal deaths, but may suggest a change in the epidemiological profile of MM depicted by a rise in the number of deaths occurring after the 42-days postpartum reference period [5, 7, 18, 20].

The analyses presented in this study highlight the rise in late maternal deaths (O96) and deaths from sequelae of obstetric causes (O97) in the Americas this past decade. Our results suggest a significant rise in the percentage of these deaths from 3.7% to 8.7% and a risk of these deaths that is 2.4 times higher in 2006–2013 compared to 1999–2005, confirming that late maternal deaths are more frequently recorded in death certificates of countries included in this study, either due to the fact that deaths certificates were modified to improve the capture of deaths occurring after 42 days postpartum or because women are surviving longer due to improvements in pregnancy-related health care.

Given the relevance and magnitude of late and obstetric sequelae maternal deaths in the region of the Americas, the authors recommend reviewing the definition of a maternal death in order to include codes 096–097 as a standard procedure in reporting maternal deaths; the definition of a maternal death ought to be reflective of the true burden of pregnancy-related complications, as lack of awareness about late maternal mortality leads to fragmented care and missed opportunities during pregnancy and after delivery [17].

The underlying cause of these deaths can be better understood if countries investigate the cases and classify them according to the additional information as direct or indirect late and obstetric sequelae maternal deaths, which may largely drive the increased number of maternal deaths coded as late or from sequelae of obstetric causes.

Much progress has been made in the information systems at the country level, enabling more effective surveillance, intentional search, and reclassification of maternal deaths, thus allocating a larger number of deaths in the late and sequelae categories defined in the ICD-10 [5, 18, 20]. For instance, the insertion of a specific question regarding pregnancy status on death certificates could explain the apparent upward trend of the MMR in the United States [15]. In Mexico, nation-wide intentional search and reclassification of maternal deaths was implemented in 2003 [18], therefore the larger number of deaths classified as late or from obstetric sequelae post-2005 is likely to be associated with stronger efforts to search for cause-specific maternal deaths. Consequently, the combination of availability of better tools for classification such as the establishment of maternal deaths committees and intentional search of these deaths in countries such as the USA, Mexico and Brazil, in addition to stronger national information systems may be the root of the recent surge in maternal deaths coded O96-O97 in the Americas this past decade.

An argument could be made about a possible epidemiological shift in the timing of maternal deaths and its associated risk: with increased access to modern care, several studies have hypothesized that improvements in maternal care may be leading to delayed maternal deaths and longer pregnancy-complication survival [21, 22]; however, our data does not allow to support this claim, and more robust investigations are needed.

Although the 2014 MMEIG estimates included these deaths in the calculation of their estimates, it is worth noting that for countries such as Brazil and USA, the MMR estimates may be significantly different from the MMR when excluding O96-O97 codes reported by the countries. At a crucial time for adherence to the SDGs, country data ownership and monitoring are essential, and therefore maximum concurrence between definitions, estimates, and countries’ epidemiological profiles will be fundamental. Due to the exclusion of late maternal deaths in the 2015 estimations [3, 4] a reduction in MMR was reported, particularly for the countries of the Americas where our data indicate proportions of O96-O97 deaths ranging between 2.40% and 18.68%, which are significantly higher than those estimated by the MMEIG in 2014 [11]. The MMEIG’s 2015 estimates publication refers to this recent rise as part of the “obstetric transition”, and urge for further analyses and monitoring of late maternal deaths in order to investigate the changing trends of maternal health [3, 4]. Nevertheless, it is important to recognize that including late and obstetric sequelae maternal deaths in the monitoring of the SDGs could be an added load to countries with already weakened health information systems, in response to the increased recording of these deaths in the Americas.

Various limitations were encountered in our evaluation. The data reported in our paper does not necessarily match countries’ reported data: for instance, country- reported data may include deaths due to HIV (code O98.7) as per PAHO’s regional mortality database, although not all countries in the region are implementing this code, and code A34 of obstetrical tetanus as recommended in ICD-10 volume 2; these may contribute to an increased MMR. Additionally, the denominator used for the calculation of ratios of late and obstetric sequelae maternal deaths in this analysis were the live births estimates by the United Nations, which does not necessarily match with countries’ reported live births. Nonetheless, we used the most recent 2015 UN estimates in order to take into account changing fertility trends.

## Recommendations

Given the upward trend in the number of ascertained late maternal deaths and deaths from obstetric sequelae in the Americas, this study serves as a call for more in depth investigation on the inherent cause of the rise in maternal deaths occurring after the 42 days postpartum reference period. Particularly, the ongoing revision process for ICD-11 is an opportunity to improve the definition and classification of maternal deaths with the aim to reflect the changing burden of maternal mortality.

Although our study focused on a trend analysis of late maternal deaths and deaths from sequelae of obstetric causes in seven countries of the Americas, it is critical to realize that misclassification and under-reporting of indirect obstetric causes of maternal deaths contribute to a larger number of all maternal deaths, once the quality has been improved [18]; therefore, the revision process for ICD-11 ought to consider improvements to the entire chapter XV, with inputs from experts from various disciplines to address the challenges in classifying maternal deaths, and ultimately, to consider all epidemiological aspects of maternal mortality and implement appropriate strategies to reduce these deaths.

As we transition into the SDGs, country-ownership in terms of measurement expertise and improved information systems will be crucial for monitoring reductions in maternal mortality [23], including late maternal deaths and deaths from obstetric sequelae. Reporting of deaths is a means to measure the advances—or lack thereof- of the actions countries take to reduce maternal mortality. The analysis of the causes, such as the ones here presented which point towards later deaths, should guide health services to review the guidelines and enhance postpartum care in order to detect early signs of health deterioration that may result in a maternal death. This way, services will have a preventive opportunity to diminish late maternal deaths.
